# Time-resolved phenotyping of seizure evolution in developmental and epileptic encephalopathy: insights and gaps from the CYFIP2 Arg87Cys mouse model

**DOI:** 10.3389/fnsyn.2026.1963161

**Published:** 2026-09-24

**Authors:** Ruiying Ma, U Suk Kim, Yousun Chung, Kihoon Han

**Affiliations:** 1 Department of Neuroscience, Korea University College of Medicine, Seoul, Republic of Korea; 2 BK21 Graduate Program, Department of Biomedical Sciences, Korea University College of Medicine, Seoul, Republic of Korea; 3 Department of Laboratory Medicine, Kangdong Sacred Heart Hospital, Seoul, Republic of Korea; 4 KUNIST Institute for Medical Science and Technology, Korea University, Seoul, Republic of Korea

**Keywords:** CYFIP2, developmental and epileptic encephalopathy, gliosis, lipid droplets, seizure evolution, synaptic remodeling, time-resolved multi-omics, West syndrome

## Abstract

In developmental and epileptic encephalopathies (DEEs), the seizure phenotype is rarely static. Infantile epileptic spasms typically subside and are often replaced years later by other seizure types, and in several genetically defined DEEs seizure-free intervals precede seizure recurrence. Although these transitions are linked to long-term outcome, little is known about the molecular and cellular events that accompany them, because the interval is clinically silent and brain tissue is inaccessible. Animal studies have only partly filled this gap: most DEE models have been characterized at a single age, and models with continuous seizures provide no interval to investigate. We recently performed time-resolved deep phenotyping of *Cyfip2^+/R87C^* mice, which recapitulate neonatal seizure-like events, a prolonged seizure-free interval, and adult-onset spontaneous recurrent seizures with premature death, using transcriptomic, ultrastructural, proteomic, and lipidomic analyses across four postnatal stages. Here we summarize the temporal landscape: excitatory synapse number falls while remaining synapses relocate to dendritic shafts, oligodendrocyte-lineage cells, microglia, and astrocytes are engaged sequentially, and lipid droplets accumulate progressively in astrocytes. We also highlight what remains unresolved. The highest-resolution analyses were performed only after seizure recurrence, illustrating a tradeoff between analytical depth and temporal coverage, so the juvenile interval preceding recurrence remains the central knowledge gap. Even so, the emerging picture is that the seizure-free interval is not a period of quiescence but one of active neuron–glia and metabolic remodeling. Defining this interval more precisely, and identifying biomarkers for patient staging, may transform a clinically silent phase into a well-defined therapeutic window for stage-specific intervention.

## 1 Introduction

Developmental and epileptic encephalopathies (DEEs) represent the most severe group of epilepsies, presenting in infancy or childhood with seizures, developmental regression, and increased mortality. The number of monogenic epilepsy genes has expanded rapidly, with the Genes4Epilepsy resource now cataloging over 900 genes, the majority of which are associated with DEEs (Oliver et al., 2023; Scheffer et al., 2024). One feature of these disorders is clinically familiar yet mechanistically almost unexamined, namely that the seizure phenotype changes over time and that these changes do not follow a simple monotonic course.

West syndrome provides the clearest illustration. Infantile spasms generally remit, yet up to 60% of patients subsequently develop other seizure types, including Lennox–Gastaut syndrome (LGS), and this progression is closely associated with poor outcome (Sillanpaa et al., 2016; Pavone et al., 2020). Between 20 and 40% of children with LGS have a preceding history of infantile epileptic spasms syndrome, and approximately one quarter to one third of children with spasms eventually evolve to LGS (Nelson et al., 2021; Deering et al., 2024). Importantly, this evolution is frequently not continuous. In a recent multicenter cohort, two thirds of children who developed LGS had passed through a seizure-free interval, or gap, between the resolution of early-life epilepsy and the emergence of the later syndrome, and slow spike–wave activity often appeared on the electroencephalogram before or during this interval (Berg et al., 2018; Deering et al., 2024). Comparable intervals occur in other genetically defined DEEs, since 38% of adults with KCNQ2 encephalopathy have experienced seizure-free periods of at least 2 years followed by recurrence (Boets et al., 2022), and the honeymoon period of CDKL5 deficiency disorder typically separates recognized stages of the epilepsy (Bahi-Buisson et al., 2008; Amin et al., 2022).

The therapeutic implication has been stated explicitly for the LGS gap, in that the interval during which the network is reorganized should also be the interval during which intervention is most likely to alter the trajectory (Berg et al., 2018). The obstacle is equally clear, since the child appears improved, brain tissue is inaccessible, and there is no means of determining whether a patient is progressing toward a more severe syndrome or has stabilized.

Animal models should in principle address questions that cannot be approached in patients, but two constraints have been limiting. First, most mechanistic studies of DEE models have sampled a single age, usually that of the most pronounced phenotype, and such designs cannot by their nature reveal sequence. Second, the models most widely used to study West syndrome, namely those carrying *Arx* or *Stxbp1* mutations, seize at relatively early developmental stages and have been examined principally in terms of interneuron migration and GABAergic transmission (Price et al., 2009; Kovacevic et al., 2018; Chen et al., 2020), so that they offer neither a prolonged interval to interrogate nor a systematic assessment of glial and metabolic change.

We recently addressed these limitations using *Cyfip2^+/R87C^* mice, which carry the recurrent p.Arg87Cys variant identified in patients with West syndrome and reproduce a complete seizure evolution, consisting of neonatal spasm-like behavioral events, a seizure-free juvenile period, spontaneous recurrent seizures from around postnatal week (PWK) 14, and premature death, and which we followed using bulk and single-nucleus transcriptomics, ultrastructural analysis, proteomics, and lipidomics (Kang et al., 2023; Ma et al., 2025). Because these events were not confirmed by electroencephalography (EEG), we refer to them throughout as seizure-like events rather than as spasms, which are defined electroclinically. To our knowledge this is the first multi-omic profiling across multiple stages of seizure evolution in a West syndrome model, and it is at the same time incomplete in an instructive way, in that its highest-resolution measurements were clustered after seizures had already begun. The present review summarizes what this sampling revealed, states which conclusions the design supports, and proposes a study that could address the remaining gap (Figure 1). Throughout, we treat the poorly characterized juvenile/young adult interval as the central gap, and separate findings obtained directly in these mice from mechanisms inferred from wider CYFIP literature.

**Figure 1:**
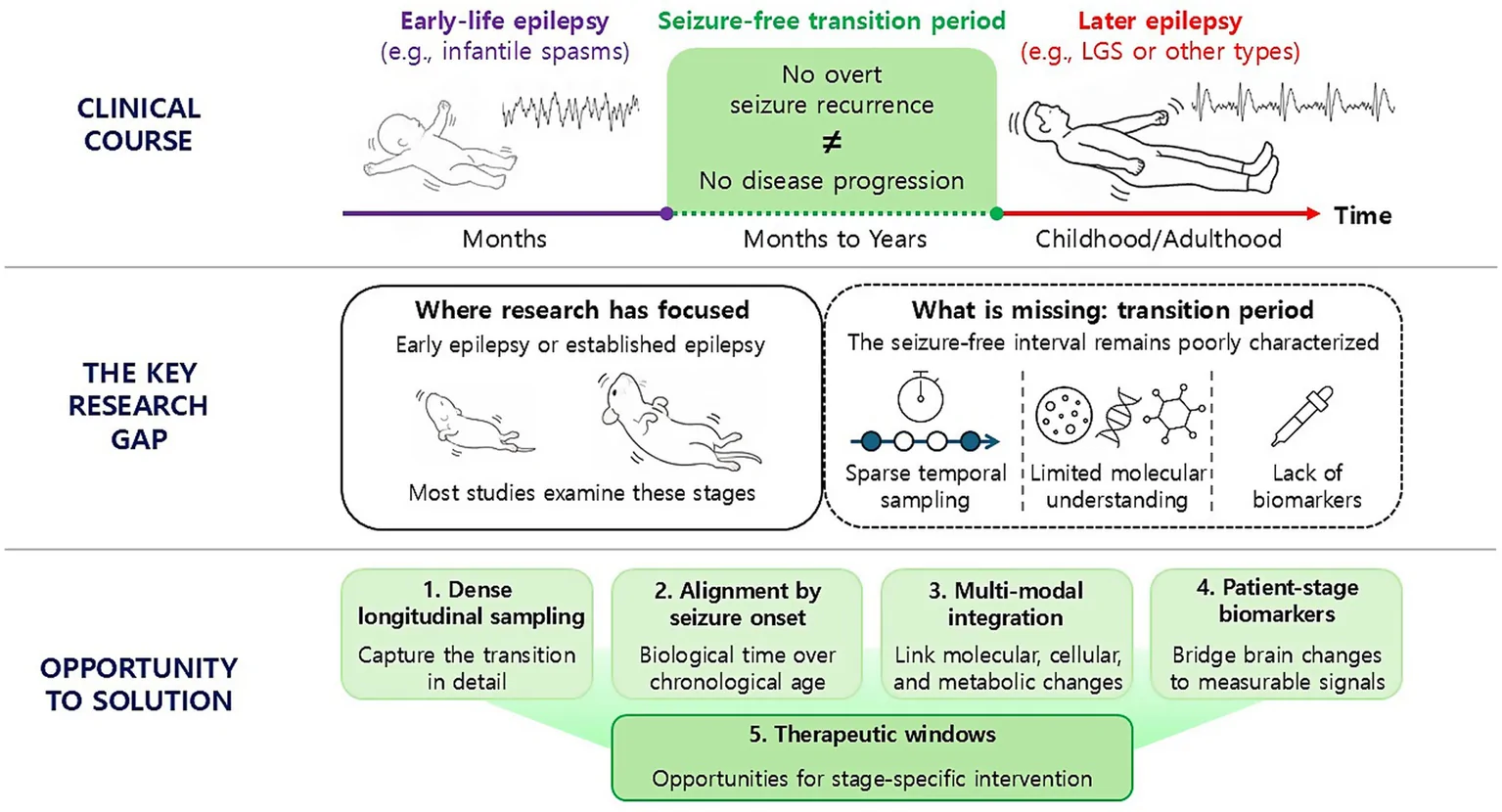
The seizure-free interval in developmental and epileptic encephalopathies: a clinically silent phase, an experimental blind spot, and an opportunity for stage-specific intervention. Top panel: In many DEEs the seizure phenotype evolves rather than remaining static. Early-life epilepsy, such as the infantile spasms of West syndrome, is followed in a substantial proportion of patients by a seizure-free interval lasting months to years, and subsequently by the emergence of other seizure types, including Lennox–Gastaut syndrome (LGS), during childhood or adulthood. Critically, the absence of overt seizure recurrence during this interval does not imply the absence of disease progression. Middle panel: Most experimental and clinical studies have examined either early-life epilepsy or established epilepsy, so that the intervening transition period remains poorly characterized. Several limitations converge on this stage: temporal sampling is sparse, particularly for high-resolution assays; neuronal and circuit function has not been characterized; the molecular and cellular processes operating during the interval are largely unknown; and no validated biomarkers exist by which the stage can be identified in an individual patient. Bottom panel: A stepwise path from this gap toward a solution. Dense longitudinal sampling would capture the transition in detail (1), and aligning animals by individual seizure onset would place events on a biological rather than a chronological timescale (2). Integrating molecular, cellular, and metabolic measurements (3), and bridging the resulting brain changes to signals measurable in patients such as fluid, imaging, and electrophysiological markers (4), would together define a therapeutic window for stage-specific intervention (5).

## 2 Seizure-free intervals in DEE and the classical latent period

Three points frame the biology discussed below. First, a seizure-free interval is a feature of some DEEs, not a defining property of the group. Dravet syndrome and epilepsy of infancy with migrating focal seizures generally show persistent epilepsy without a prolonged seizure-free interval preceding later seizure recurrence (Wirrell et al., 2017; Scheffer et al., 2024). What distinguishes syndromes with such an interval from those without remains unknown, and the question constrains experimental design, because a model without an interval cannot be used to study one. Second, the *Cyfip2^+/R87C^* line is unusually suitable for this purpose. Its seizure-free period spans the juvenile and young adult stages, is long enough to sample repeatedly, and ends with a clearly defined onset.

Third, this interval should not be equated with the latent period of acquired epilepsy, which denotes a seizure-free, pre-epileptic state between a discrete insult and the first spontaneous seizure (Loscher et al., 2015). In acquired epilepsy, epileptogenesis and sometimes subclinical epileptic activity begin immediately after the injury, and the apparent latent period has accordingly been redescribed as a period of epileptic maturation (Sloviter and Bumanglag, 2013). Genetic DEE differs on three counts: there is no antecedent insult, the causal variant acts throughout, and seizures precede rather than follow the interval, which is therefore better described as a temporary remission within an ongoing genetic condition. One element of the debate nevertheless translates directly, namely whether the interval is genuinely quiet or merely clinically silent. The LGS literature provides a partial answer, as slow spike–wave activity can emerge before clinical seizures recur (Deering et al., 2024). Whether this clinically silent phase also has a cellular correlate is the question the mouse data considered below are best placed to address, and the question around which we have organized them.

## 3 Temporally dynamic changes in the brain of *Cyfip2^+/R87C^* mice

### 3.1 Excitatory synapses are relocated rather than simply lost

One of the more unexpected structural observations in *Cyfip2^+/R87C^* mice is that the number of excitatory synapses in the hippocampal CA1 region decreases at PWK 14 and 28 while inhibitory synapse number is preserved (Ma et al., 2025), despite the emergence of spontaneous recurrent seizures. Structural synapse counts alone, however, do not predict network excitability, since they carry no information about synaptic strength, inhibitory efficacy, or circuit organization. Increased inhibition can itself be epileptogenic, as enhanced tonic GABAergic inhibition in typical absence epilepsy models illustrates (Cope et al., 2009), and this observation therefore requires functional assessment before it can be interpreted.

Ultrastructural analysis suggests one hypothesis that could account for this discrepancy. Dendritic spines are markedly reduced, and the remaining excitatory synapses are formed predominantly on dendritic shafts, with multiple presynaptic boutons frequently converging onto individual shafts, while the distribution of postsynaptic density surface area differs from wild type (Ma et al., 2025). Since the narrow neck of a spine compartmentalizes biochemical and electrical signaling (Adrian et al., 2014), shaft synapses bypass this barrier, and such an arrangement could facilitate more effective integration of convergent excitatory inputs than would be achieved by a larger number of spatially isolated spine synapses. This structural reorganization is accompanied by axonal hypermyelination, a change for which there is precedent in other epilepsy models where maladaptive myelination is thought to facilitate seizure propagation (Knowles et al., 2022). Together, these findings suggest that excitatory circuitry is qualitatively remodeled rather than simply lost, such that fewer synapses may nonetheless support more efficient synchronization of neuronal activity. We emphasize, however, that the data do not demonstrate stronger transmission, better input integration, or enhanced synchronization; this account is a hypothesis awaiting functional testing rather than a resolution of the paradox.

This structural remodeling is accompanied by asymmetric molecular changes, in that postsynaptic markers including Shank3, Homer1, and GluA2 are decreased whereas presynaptic markers such as Synaptotagmin-1 and Synapsin 1 are increased (Ma et al., 2025). Altered exon skipping was also detected in *Nrxn2* at PWK 14 and in *Nrxn3* at PWK 28, and given that neurexin isoform choice is activity-regulated and instructive for synapse specification (Gomez et al., 2021), these events may contribute to the remodeling, although whether these splicing changes actively drive the remodeling process or instead arise as a consequence of altered neuronal activity remains unresolved.

### 3.2 Glial changes follow an apparent temporal sequence

Immunohistochemical analysis across PWK 1, 7, 14, and 28, comparing knock-in mice with age-matched wild-type littermates, suggested a temporal sequence in the mutant hippocampus, in which the number of Olig2-positive oligodendrocyte-lineage cells increased first at PWK 7 and persisted thereafter, followed by increases in IBA1 intensity at PWK 14 and GFAP intensity at both PWK 14 and 28 (Ma et al., 2025). Gene set enrichment analysis of the bulk transcriptome showed peak enrichment of cell-type-specific gene sets in the same order. At PWK 14 and 28, microglia in knock-in mice exhibited reduced Sholl intersections, branch length, and branch area relative to wild-type littermates, consistent with an ameboid, phagocytic state (Vainchtein and Molofsky, 2020), together with elevated CD68 signals and increased proinflammatory cytokine transcripts at PWK 14. Astrocytes likewise displayed simplified morphology, consistent with reactive remodeling.

This ordering is broadly consistent with findings in other systems. In a drug-induced status epilepticus model, prior activation of microglia was required for subsequent reactive astrogliosis and epileptogenesis, and pharmacological inhibition of microglial activation prevented both (Sano et al., 2021). Involvement of the oligodendrocyte lineage also has support, as increased numbers of Olig2-positive cells have been reported in epilepsy models and in human epileptic brain tissue (Luo et al., 2015; Sone et al., 2018), and oligodendrocyte precursor cells engulf presynaptic material during activity-dependent circuit remodeling in the developing brain (Auguste et al., 2022). Whereas Sano et al. (2021) demonstrated a microglia-to-astrocyte sequence following status epilepticus, our data raise the possibility that changes within the oligodendrocyte lineage may precede both microglial and astrocytic activation in this genetic model. The particular value of the *Cyfip2^+/R87C^* data is that it places all three glial lineages on a single temporal framework within the same disease model, enabling their relative timing to be compared directly rather than inferred across separate studies.

An important caveat, however, is that PWK 7, 14, and 28 are widely spaced, so that the relative order of microglial and astrocytic engagement should be regarded as suggested rather than established. Moreover, because Olig2 labels both oligodendrocyte precursor cells and differentiated oligodendrocytes, the present data cannot determine which oligodendrocyte-lineage population initiates the earliest response. Resolving this question will require additional lineage-specific markers and finer temporal sampling. Because CYFIP2 is expressed predominantly in neurons (Ma et al., 2022), these glial changes are also unlikely to be cell-autonomous consequences of the variant. The IBA1 and GFAP changes coincide with seizure onset and may be reactive rather than contributory, and the earlier Olig2 increase may be a delayed response to the neonatal events. The ordering is therefore a temporal association rather than a causal cascade.

### 3.3 Lipid and mitochondrial alterations emerge at a late stage

Electron microscopy of hippocampal astrocytes at PWK 28 revealed enlarged lipid droplets within lysosomes, together with crystalline material on and within the droplets that was never observed in wild-type mice, whereas neither the number nor the volume of lipid droplets differed between genotypes at PWK 14 (Ma et al., 2025). Astrocytic mitochondria at PWK 28 were also smaller and lacked distinct cristae. Multiple independent datasets converged on the same conclusion. Single-nucleus transcriptomics identified an astrocyte subtype that was more abundant in mutant samples and showed elevated *Apoe* and *Gfap* expression together with enrichment of oxidative phosphorylation and fatty acid metabolism pathways. In parallel, *Apod* and *Ptgds* were among the most prominently upregulated genes and were confirmed at the protein level, while lipidomic profiling demonstrated increased triacylglycerol, diacylglycerol, and cholesteryl ester, the major lipid classes found in lipid droplets (Zadoorian et al., 2023; Ma et al., 2025).

These observations fit well within an emerging framework in which hyperactive neurons transfer excess fatty acids to astrocytes for oxidation (Ioannou et al., 2019), while lipid-accumulated reactive astrocytes identified in temporal lobe epilepsy exhibit impaired glutamate uptake and promote disease progression (Chen et al., 2023). Accordingly, our findings are consistent with a sequential cascade in which recurrent seizures increase lipid flux toward astrocytes whose mitochondrial capacity is already compromised, thereby promoting lipid accumulation. Whether lipid-laden astrocytes in turn enhance neuronal hyperexcitability, which would close this sequence in a feed-forward cycle, is not established in the present model: the return arm is inferred from temporal lobe epilepsy tissue rather than demonstrated here, and the intervening steps linking lipid accumulation, fatty acid oxidation, and glial activation to neuronal dysfunction remain untested. We therefore describe an interconnected cascade rather than a feed-forward loop. This account remains hypothetical, resting on correlative observations from a single late disease stage. The opposite direction of causality is equally consistent with the data, since these abnormalities may be downstream consequences of weeks of recurrent seizures, in which case they would mark cumulative seizure burden rather than drive progression. Seizure suppression experiments would be required to distinguish the two.

## 4 Limitations of the sampling design: temporal coverage and functional characterization

The value of an age-resolved design lies in its ability to define temporal sequence, and it is therefore worth considering which stages were actually sampled by each analysis. Bulk transcriptomics, quantitative RT-PCR, Western blotting, and immunohistochemistry for neuronal and glial markers were performed at PWK 1, 7, 14, and 28. By contrast, immunohistochemical quantification of synapses and glial morphology, proteomics, and lipidomics were performed only at PWK 14 and 28, lipid droplet analysis at PWK 14 and 28, electron microscopy and single-nucleus transcriptomics only at PWK 28, and video-EEG recordings only at PWK 20 from three animals. Two general patterns emerge. First, the higher the resolution of an assay, the fewer the sampled time points. Second, the critical transition between PWK 7 and PWK 14 was not sampled directly. The terminal analyses were also performed in separate cohorts rather than repeatedly in the same animals, so the design is age-resolved rather than longitudinal in the strict sense.

Several consequences follow. The only molecular datasets spanning the pre-onset period are the bulk transcriptome and marker immunohistochemistry, and the numbers of differentially expressed genes in the hippocampus were 20, 41, and 3,755 at PWK 1, 7, and 14, respectively (Ma et al., 2025). This suggests that molecular alterations remain close to the threshold of detection before seizure onset, with a sharp increase occurring around the time seizures emerge. The elevated Olig2 signal at PWK 7 therefore represents the strongest cellular evidence of change during the seizure-free interval, whereas observations on synaptic ultrastructure, astrocytic lipid handling, mitochondrial morphology, and the proteome and lipidome all describe a brain in which spontaneous seizures are already established. Moreover, because perioperative mortality approached 80%, video-EEG recordings were restricted to a single time point and only three animals, leaving behavioral seizure scoring as the primary measure of disease progression.

This pattern is not unique to the present study. Deep molecular analyses are terminal, tissue-intensive, and expensive, so analytical depth is often gained at the expense of temporal resolution, whereas broad temporal coverage is generally achievable only with lower-resolution assays. As a result, the stage of disease that is arguably most important—the clinically silent interval—is also the one that remains least well characterized (Figure 1, middle panel).

A second and more consequential limitation concerns electrophysiology. The neonatal events were scored behaviorally and not confirmed by EEG, and no intracranial recording was obtained at any juvenile age, although such recordings are feasible in mouse pups following recovery from anesthesia (Dominguez et al., 2021) and have been applied to other DEE models (Teoh et al., 2025). Neuronal and circuit function was likewise not examined across the neonatal seizure phase or the juvenile interval: intrinsic excitability and action-potential firing, spontaneous and miniature excitatory and inhibitory postsynaptic currents, evoked transmission, and extracellular network activity are all absent from this window. The interval is therefore seizure-free by behavioral criteria alone, and interictal discharges or altered excitability during it cannot be excluded. Of the gaps identified here, we would place this one first.

## 5 Challenges in distinguishing seizure-driven from variant-driven changes

If most of the deep phenotyping describes a brain that is already seizing, an obvious question is to what extent the observed changes are downstream of seizures rather than upstream of them, since recurrent seizures themselves are capable of remodeling synapses, activating glial cells, and imposing a substantial metabolic burden (Patel et al., 2019; Rho and Boison, 2022).

The study contains one useful internal control. *Cyfip2^+/−^* mice, in which CYFIP2 protein levels are likewise reduced, show no lethality, no spontaneous recurrent seizures, essentially normal hippocampal cytoarchitecture, and only mild GFAP elevation, and their transcriptomic signature remains remarkably limited, with only 19 differentially expressed genes in the hippocampus at PWK 14 compared with 3,755 in the knock-in line (Ma et al., 2025). Whatever produces the phenotype in *Cyfip2^+/R87C^* mice is therefore unlikely to reflect a simple reduction in gene dosage, consistent instead with a toxic gain-of-function effect of the Arg87 hotspot variant.

An additional consideration is that CYFIP2 is expressed predominantly in neurons, with relatively low expression in glial cells (Ma et al., 2022). Accordingly, many of the glial abnormalities observed in the knock-in mice are unlikely to represent direct cell-autonomous effects of the variant, but instead probably arise through altered neuronal activity, recurrent seizures, or secondary neuron–glia interactions.

This comparison does not, however, separate seizure-driven from variant-driven changes within the knock-in line itself, and two observations provide partial clues. Astrocytic lipid droplet changes were absent at PWK 14 but prominent at PWK 28, suggesting that they accumulate with disease progression and increasing seizure burden rather than accompanying seizure onset. By contrast, the increase in Olig2-positive cells at PWK 7 occurred during the seizure-free interval and therefore cannot be explained by ongoing spontaneous seizures, although it could still reflect a delayed response to the neonatal seizure-like events.

Because direct evidence from within the interval is limited, candidate variant-driven mechanisms must for now be inferred from the wider CYFIP literature. CYFIP2 is a subunit of the WAVE regulatory complex, which controls Arp2/3-dependent actin branching and spine morphogenesis, and variants clustering around Arg87 are thought to destabilize its autoinhibited conformation (Zweier et al., 2019), placing actin regulation upstream of the spine loss described above. CYFIP2 also represses cap-dependent translation through eIF4E and FMRP (Ghosh et al., 2020). Whether the Arg87Cys variant alters this function has not been tested. The interval is moreover when excitatory and inhibitory synapses mature, so a variant acting on synapse formation would exert its largest effect precisely where sampling is thinnest. None of these mechanisms has been tested in these mice, and we raise them as hypotheses rather than conclusions.

## 6 Multiple therapeutic windows and the problem of staging

Considered as a whole, the temporal data imply that different classes of intervention would be most effective at different stages of disease. Allele-selective suppression of the mutant transcript, for instance using antisense oligonucleotides (Mittal et al., 2022), would likely be most effective early, before glial engagement becomes prominent, since CYFIP2 is expressed predominantly in neurons rather than glia. Approaches targeting glial activation and inflammatory signaling would fit an intermediate stage, whereas interventions aimed at restoring lipid and metabolic homeostasis, such as liver-X receptor agonism (Litvinchuk et al., 2024), may be more appropriate later. The principal contribution of age-resolved studies is therefore not to identify a single therapy, but to show that multiple therapeutic windows likely exist, each governed by different biology.

The greatest challenge is that a therapeutic window is useful only if it can be identified prospectively, yet no validated staging biomarkers currently exist for DEEs. Electrophysiological markers provide the strongest precedent, as slow spike–wave activity preceding seizure recurrence in children who later develop LGS may herald progression (Deering et al., 2024). Imaging and fluid biomarkers may complement this approach. Hippocampal abnormalities and lipid accumulation could potentially be monitored by magnetic resonance imaging and spectroscopy (Chen et al., 2023), while plasma GFAP and neurofilament light chain represent promising fluid biomarkers (Thaele et al., 2025), and PTGDS and APOD emerge from the present model as candidate staging markers, although their utility for staging remains to be established.

## 7 Discussion

Time-resolved phenotyping of *Cyfip2^+/R87C^* mice indicates that seizure evolution in a West syndrome model is accompanied by reorganization, rather than simple loss, of excitatory synapses, sequential engagement of three glial lineages, and progressive lipid and mitochondrial abnormalities in astrocytes. Bulk transcriptomic and proteomic analyses further revealed progressive downregulation of mRNA splicing and processing, potentially reflecting both altered CYFIP2–RNA-binding protein interactions (Zhang et al., 2024) and secondary effects of seizure activity. However, because the deepest analyses were performed only after seizure recurrence, the transition from the neonatal seizure-like events to adult seizures remains largely uncharacterized.

Several directions follow (Figure 1, bottom panel). First, the transition period should be sampled more densely, with animals aligned by seizure onset rather than chronological age. Continuous EEG spanning this interval would establish individual onset times and reduce between-animal variability. Functional measurements within the interval, including intrinsic excitability and synaptic transmission, should accompany it. Cell-resolved methods, including single-nucleus and spatial transcriptomics, could determine whether the near-silent bulk transcriptomic signal conceals changes within specific cell populations. Seizure-driven and variant-driven changes should also be separated experimentally, for example by comparing pharmacologically treated and untreated knock-in mice, as changes that persist despite seizure suppression would become candidates for upstream disease mechanisms. The proposed glial sequence should be tested causally through cell-type-specific, temporally restricted manipulations, following the logic already applied in a status epilepticus model (Sano et al., 2021). Finally, applying a common onset-aligned panel of assays across *Arx*, *Stxbp1*, *Cdkl5*, and acquired epilepsy models could distinguish features specific to CYFIP2 dysfunction from general mechanisms of seizure evolution, with convergent findings representing the most promising therapeutic targets.

The next advance may come less from adding analytical modalities than from placing existing ones at the right time: sampling the transition more densely, aligning animals to biological rather than chronological time, and linking changes in the brain to biomarkers measurable in patients before seizure recurrence becomes clinically apparent.
